# Crystal structure of ethyl 6-bromo-2-[(*E*)-2-phenyl­ethen­yl]quinoline-4-carboxyl­ate

**DOI:** 10.1107/S2056989014028266

**Published:** 2015-01-17

**Authors:** T. O. Shrungesh Kumar, S. Naveen, M. N. Kumara, K. M. Mahadevan, N. K. Lokanath

**Affiliations:** a Department of Chemistry, Kuvempu University, Jnanasahyadri, Shankaraghatta 577 451, India; b Institution of Excellence, University of Mysore, Manasagangotri, Mysuru 570 006, India; c Department of Chemistry, Yuvaraja’s College, University of Mysore, Mysuru 570 005, India; d Department of Chemistry, Kuvempu University, Jnanasahyadri, Shankaraghatta 577451, India; e Department of Studies in Physics, University of Mysore, Manasagangotri, Mysuru 570 006, India

**Keywords:** crystal structure, quinoline, quinoline-4-carboxyl­ate, hydrogen bonding

## Abstract

In the title compound, C_20_H_16_BrNO_2_, the dihedral angle between the quinolone ring system mean plane (r.m.s. deviation = 0.018 Å) and the phenyl ring bridged by the ethynyl group, is 25.44 (14)°. There is an intra­molecular C—H⋯O hydrogen bond forming an *S*(6) ring motif. In the crystal, mol­ecules are linked *via* C—H⋯O hydrogen bonds forming chains propagating along the *b*-axis direction.

## Related literature   

For pharmaceutical and pharmacological activities of quinolines, see: Beagley *et al.* (2003 ▸). The title compound was synthesized in a continuation of our work on new quinoline-based therapeutic agents, see: Pradeep *et al.* (2014 ▸).
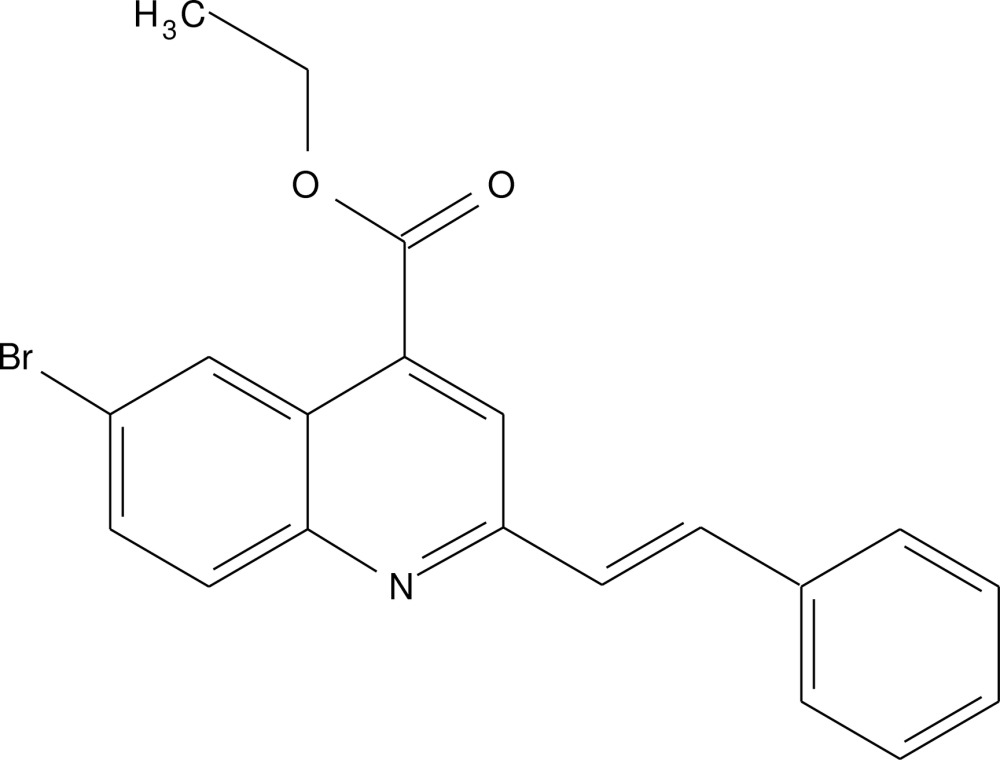


## Experimental   

### Crystal data  

C_20_H_16_BrNO_2_
*M* *_r_* = 382.24
Orthorhombic, 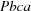

*a* = 14.0819 (7) Å
*b* = 9.7470 (5) Å
*c* = 24.0399 (12) Å
*V* = 3299.6 (3) Å^3^
*Z* = 8
Cu *K*α radiation
μ = 3.49 mm^−1^
*T* = 293 K
0.30 × 0.27 × 0.25 mm

### Data collection  

Bruker X8 Proteum diffractometer
Absorption correction: multi-scan (*SADABS*; Bruker, 2013 ▸) *T*_min_ = 0.421, *T*_max_ = 0.476
12970 measured reflections
2722 independent reflections
2213 reflections with *I* > 2σ(*I*)
*R*_int_ = 0.071

### Refinement  

*R*[*F* ^2^ > 2σ(*F* ^2^)] = 0.046
*wR*(*F* ^2^) = 0.134
*S* = 1.04
2722 reflections
218 parameters
H-atom parameters constrained
Δρ_max_ = 0.78 e Å^−3^
Δρ_min_ = −0.92 e Å^−3^

### 

Data collection: *APEX2* (Bruker, 2013 ▸); cell refinement: *SAINT* (Bruker, 2013 ▸); data reduction: *SAINT*; program(s) used to solve structure: *SHELXS97* (Sheldrick, 2008 ▸); program(s) used to refine structure: *SHELXL97* (Sheldrick, 2008 ▸); molecular graphics: *Mercury* (Macrae *et al.*, 2008 ▸); software used to prepare material for publication: *SHELXL97*.

## Supplementary Material

Crystal structure: contains datablock(s) global, I. DOI: 10.1107/S2056989014028266/su5042sup1.cif

Structure factors: contains datablock(s) I. DOI: 10.1107/S2056989014028266/su5042Isup2.hkl

Supporting information file. DOI: 10.1107/S2056989014028266/su5042Isup3.cml

**Fig. 1.**
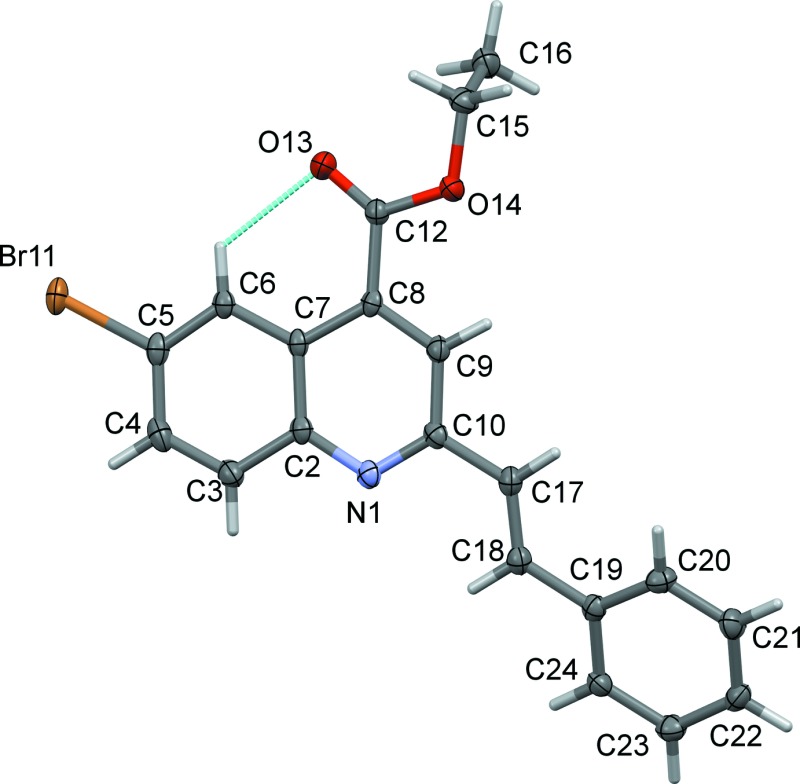
A view of molecular structure of the title compound, with atom labelling. Displacement ellipsoids are drawn at the 50% probability level. The intramolecular hydrogen bond is shown as dashed line (see Table 1 for details).

**Fig. 2.**
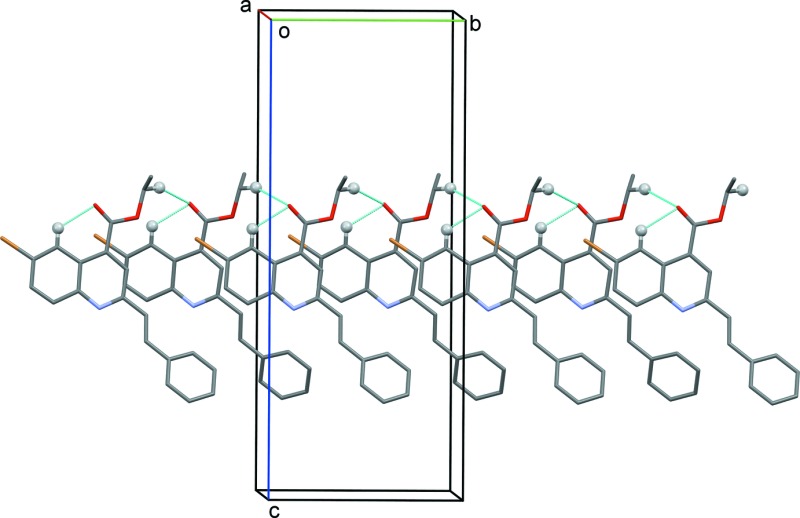
A partial view along the a axis of the crystal packing of the title compound. The intra- and inter-molecular hydrogen bonds are shown as dashed lines (see Table 1 for details; H atoms: grey balls; H atoms not involved in hydrogen bonding have been omitted for clarity).

. DOI: 10.1107/S2056989014028266/su5042fig1.tif
A view of mol­ecular structure of the title compound, with atom labelling. Displacement ellipsoids are drawn at the 50% probability level. The intra­molecular hydrogen bond is shown as dashed line (see Table 1 for details).

a . DOI: 10.1107/S2056989014028266/su5042fig2.tif
A partial view along the *a* axis of the crystal packing of the title compound. The intra- and inter-mol­ecular hydrogen bonds are shown as dashed lines (see Table 1 for details; H atoms: grey balls; H atoms not involved in hydrogen bonding have been omitted for clarity).

CCDC reference: 1041593

Additional supporting information:  crystallographic information; 3D view; checkCIF report

## Figures and Tables

**Table 1:** Hydrogen-bond geometry (, )

*D*H*A*	*D*H	H*A*	*D* *A*	*D*H*A*
C6H6O13	0.93	2.22	2.848(4)	124
C15H15*A*O13^i^	0.97	2.51	3.413(4)	154

Symmetry code: (i) 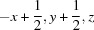.

## supplementary crystallographic information

### S1. Comment

Quinolines have been considered as the most prevalent N-hetero aromatic compounds that exhibit a wide spectrum of pharmaceutical and pharmacological activities (Beagley *et al.*, 2003). Some of the quinoline-4-carboxylates were reported to possess potent 5HT_3_ antagonizing activity and anti-emetic activity. In view of their broad spectrum of medicinal properties and in continuation of our work on new quinoline based therapeutic agents (Pradeep *et al.*, 2014), the title compound was synthesized, and we report herein on its crystal structure.

The molecular structure of the title molecule is shown in Fig. 1. The quinoline ring system (N1/C2-C10) is planar with the maximum deviations from the mean plane being for atoms C8 and C5 *viz*. 0.018 (2) Å. The dihedral angle between the quinoline ring and the phenyl ring (C19–C24) bridged by the ethynyl group is 25.44 (14)°. The two rings of the quinolyl moiety are fused in an axial fashion and form a dihedral angle of 1.15 (13)°.

In the crystal, molecules are linked via C–H···O hydrogen bonds forming chains propagating along the *b* axis direction (Table 1 and Fig. 2).

### S2. Experimental

A mixture of 2-aryl-6-chloro/bromo quinoline-4-carboxylic acid (1.0 g) and absolute EtOH (15 ml) was stirred at 273 - 278 K. The concentrated sulfuric acid (2 - 3 ml) was added drop wise into the flask until the powdered 2-aryl-6-chloroquinoline-4-carboxylic acid was completely dissolved. The solution was then refluxed for 15–17 h. The completion of the reaction was monitored by thin layer chromatography [hexane and ethyl acetate (9:1 *v*/*v*)]. The reaction mixture was poured into a crushed ice (100 ml), the precipitate was collected by filtration, washed with water and EtOH, dried under vacuum to afford crude product. The crude product was purified by column chromatography using silica gel (60–120 mesh, petroleum ether: ethyl acetate, 9:1 *v*/*v*). Green block-shaped crystals were obtained by slow evaporation of the solvent.

^1^H-NMR(400 MHz, CDCl_3_): δ = 8.95 (d, J = 2.00 Hz, 1H), 8.17 (s, 1H), 7.99 (d, J = 4.40 Hz, 1H), 7.82 (d, J = 2.40 Hz, 1H), 7.81 (t, J = 2.00 Hz, 1H), 7.75 (s, 1H), 7.65 (d, J = 7.20 Hz, 1H), 7.35–7.35 (m, 4H), 4.54 (q, J = 7.20 Hz, 2H), 1.46–1.49 (m, 3H) p.p.m.. MS (70 eV) m/*z* (%): 382.0 (*M*^+^).

### S3. Refinement

All the H atoms were fixed geometrically (C—H = 0.93–0.96 Å and allowed to ride on their parent atoms with *U*_iso_(H) = 1.5*U*_eq_(C) for methyl H atoms and = 1.2*U*_eq_(C) for other H atoms.

### Crystal data

**Table:**

C_20_H_16_BrNO_2_	*F*(000) = 1552
*M**_r_* = 382.24	*D*_x_ = 1.539 Mg m^−^^3^
Orthorhombic, *P**b**c**a*	Cu *K*α radiation, λ = 1.54178 Å
Hall symbol: -P 2ac 2ab	Cell parameters from 2722 reflections
*a* = 14.0819 (7) Å	θ = 5.8–64.5°
*b* = 9.7470 (5) Å	µ = 3.49 mm^−^^1^
*c* = 24.0399 (12) Å	*T* = 293 K
*V* = 3299.6 (3) Å^3^	Block, green
*Z* = 8	0.30 × 0.27 × 0.25 mm

### Data collection

**Table:**

Bruker X8 Proteum diffractometer	2722 independent reflections
Radiation source: Bruker MicroStar microfocus rotating anode	2213 reflections with *I* > 2σ(*I*)
Helios multilayer optics monochromator	*R*_int_ = 0.071
Detector resolution: 18.4 pixels mm^-1^	θ_max_ = 64.5°, θ_min_ = 5.8°
φ and ω scans	*h* = −16→15
Absorption correction: multi-scan (*SADABS*; Bruker, 2013)	*k* = −11→11
*T*_min_ = 0.421, *T*_max_ = 0.476	*l* = −28→27
12970 measured reflections	

### Refinement

**Table:**

Refinement on *F*^2^	Primary atom site location: structure-invariant direct methods
Least-squares matrix: full	Secondary atom site location: difference Fourier map
*R*[*F*^2^ > 2σ(*F*^2^)] = 0.046	Hydrogen site location: inferred from neighbouring sites
*wR*(*F*^2^) = 0.134	H-atom parameters constrained
*S* = 1.04	*w* = 1/[σ^2^(*F*_o_^2^) + (0.0857*P*)^2^] where *P* = (*F*_o_^2^ + 2*F*_c_^2^)/3
2722 reflections	(Δ/σ)_max_ = 0.001
218 parameters	Δρ_max_ = 0.78 e Å^−^^3^
0 restraints	Δρ_min_ = −0.92 e Å^−^^3^

### Special details

**Table:**

Geometry. Bond distances, angles *etc*. have been calculated using the rounded fractional coordinates. All su's are estimated from the variances of the (full) variance-covariance matrix. The cell e.s.d.'s are taken into account in the estimation of distances, angles and torsion angles
Refinement. Refinement on *F*^2^ for ALL reflections except those flagged by the user for potential systematic errors. Weighted *R*-factors *wR* and all goodnesses of fit *S* are based on *F*^2^, conventional *R*-factors *R* are based on *F*, with *F* set to zero for negative *F*^2^. The observed criterion of *F*^2^ > σ(*F*^2^) is used only for calculating -*R*-factor-obs *etc*. and is not relevant to the choice of reflections for refinement. *R*-factors based on *F*^2^ are statistically about twice as large as those based on *F*, and *R*-factors based on ALL data will be even larger.

### Fractional atomic coordinates and isotropic or equivalent isotropic displacement parameters (Å^2^)

**Table:**

	*x*	*y*	*z*	*U*_iso_*/*U*_eq_	
Br11	0.40232 (3)	0.12926 (3)	0.45926 (2)	0.0260 (2)	
O13	0.3502 (2)	0.6096 (2)	0.39081 (10)	0.0299 (8)	
O14	0.32650 (17)	0.8275 (2)	0.41637 (9)	0.0225 (7)	
N1	0.3740 (2)	0.6330 (3)	0.60400 (12)	0.0194 (9)	
C2	0.3819 (2)	0.5243 (3)	0.56849 (15)	0.0186 (9)	
C3	0.4009 (2)	0.3948 (4)	0.59271 (17)	0.0235 (11)	
C4	0.4086 (2)	0.2801 (4)	0.56073 (17)	0.0252 (11)	
C5	0.3971 (2)	0.2916 (3)	0.50306 (16)	0.0212 (10)	
C6	0.3799 (2)	0.4140 (3)	0.47707 (15)	0.0186 (10)	
C7	0.3720 (2)	0.5345 (3)	0.50974 (14)	0.0169 (9)	
C8	0.3536 (2)	0.6688 (3)	0.48784 (14)	0.0170 (9)	
C9	0.3445 (2)	0.7753 (3)	0.52462 (14)	0.0194 (10)	
C10	0.3557 (2)	0.7552 (3)	0.58248 (14)	0.0183 (9)	
C12	0.3436 (2)	0.6941 (3)	0.42679 (14)	0.0191 (10)	
C15	0.3129 (3)	0.8638 (3)	0.35775 (15)	0.0256 (11)	
C16	0.4064 (3)	0.8926 (4)	0.33011 (17)	0.0305 (11)	
C17	0.3493 (2)	0.8754 (3)	0.61965 (15)	0.0208 (10)	
C18	0.3704 (3)	0.8739 (3)	0.67333 (15)	0.0208 (11)	
C19	0.3718 (2)	0.9924 (3)	0.71107 (14)	0.0189 (9)	
C20	0.3281 (3)	1.1179 (3)	0.69823 (15)	0.0237 (11)	
C21	0.3389 (3)	1.2296 (3)	0.73304 (16)	0.0251 (10)	
C22	0.3913 (2)	1.2187 (4)	0.78153 (15)	0.0237 (11)	
C23	0.4324 (3)	1.0941 (4)	0.79619 (15)	0.0246 (10)	
C24	0.4218 (3)	0.9823 (3)	0.76129 (14)	0.0216 (10)	
H3	0.40820	0.38790	0.63110	0.0280*	
H4	0.42130	0.19540	0.57690	0.0300*	
H6	0.37340	0.41800	0.43860	0.0220*	
H9	0.33080	0.86260	0.51130	0.0230*	
H15A	0.27260	0.94430	0.35520	0.0310*	
H15B	0.28130	0.78890	0.33870	0.0310*	
H16A	0.43890	0.96390	0.35000	0.0460*	
H16B	0.39560	0.92140	0.29240	0.0460*	
H16C	0.44440	0.81090	0.33020	0.0460*	
H17	0.32900	0.95800	0.60430	0.0250*	
H18	0.38580	0.78920	0.68870	0.0250*	
H20	0.29170	1.12590	0.66610	0.0280*	
H21	0.31060	1.31280	0.72380	0.0300*	
H22	0.39920	1.29490	0.80430	0.0290*	
H23	0.46650	1.08590	0.82910	0.0300*	
H24	0.44850	0.89860	0.77140	0.0260*	

### Atomic displacement parameters (Å^2^)

**Table:**

	*U*^11^	*U*^22^	*U*^33^	*U*^12^	*U*^13^	*U*^23^
Br11	0.0262 (3)	0.0137 (3)	0.0382 (3)	0.0007 (1)	−0.0011 (2)	−0.0051 (1)
O13	0.0497 (17)	0.0187 (12)	0.0212 (14)	−0.0009 (11)	0.0006 (12)	−0.0020 (10)
O14	0.0314 (13)	0.0173 (11)	0.0189 (12)	0.0030 (10)	0.0002 (10)	0.0021 (10)
N1	0.0182 (14)	0.0181 (15)	0.0218 (16)	−0.0046 (10)	0.0005 (12)	−0.0002 (11)
C2	0.0163 (15)	0.0166 (16)	0.0229 (18)	−0.0042 (13)	0.0002 (14)	0.0001 (14)
C3	0.026 (2)	0.0204 (18)	0.024 (2)	−0.0024 (13)	−0.0019 (14)	0.0032 (15)
C4	0.0229 (19)	0.0166 (18)	0.036 (2)	−0.0019 (13)	−0.0029 (15)	0.0059 (16)
C5	0.0158 (17)	0.0159 (17)	0.032 (2)	−0.0005 (12)	0.0000 (13)	−0.0022 (15)
C6	0.0162 (16)	0.0160 (16)	0.0236 (18)	−0.0038 (13)	0.0008 (14)	−0.0003 (14)
C7	0.0136 (15)	0.0133 (16)	0.0239 (18)	−0.0019 (13)	0.0010 (13)	−0.0003 (13)
C8	0.0140 (15)	0.0157 (15)	0.0213 (18)	−0.0035 (13)	−0.0005 (13)	−0.0023 (13)
C9	0.0208 (17)	0.0154 (16)	0.0220 (18)	−0.0019 (13)	−0.0015 (14)	−0.0006 (13)
C10	0.0157 (16)	0.0182 (16)	0.0211 (17)	−0.0029 (13)	−0.0004 (13)	0.0018 (13)
C12	0.0202 (17)	0.0158 (15)	0.0212 (18)	−0.0003 (13)	0.0009 (13)	−0.0006 (14)
C15	0.032 (2)	0.0246 (18)	0.0201 (19)	0.0066 (14)	−0.0022 (15)	0.0029 (14)
C16	0.043 (2)	0.0216 (18)	0.027 (2)	−0.0002 (15)	0.0061 (16)	0.0010 (16)
C17	0.0218 (17)	0.0154 (16)	0.0253 (19)	−0.0014 (13)	0.0009 (14)	−0.0010 (13)
C18	0.0209 (18)	0.0174 (17)	0.024 (2)	−0.0010 (13)	0.0021 (14)	0.0001 (13)
C19	0.0180 (15)	0.0179 (15)	0.0207 (17)	−0.0031 (13)	0.0030 (14)	−0.0007 (14)
C20	0.0236 (19)	0.0276 (18)	0.0200 (19)	0.0020 (14)	−0.0020 (14)	−0.0011 (14)
C21	0.0296 (19)	0.0190 (16)	0.0267 (19)	0.0063 (14)	0.0016 (15)	0.0004 (15)
C22	0.0267 (19)	0.0232 (17)	0.0213 (19)	−0.0062 (14)	0.0043 (14)	−0.0063 (14)
C23	0.0257 (18)	0.0279 (17)	0.0203 (18)	−0.0015 (15)	−0.0031 (15)	0.0007 (15)
C24	0.0264 (18)	0.0189 (16)	0.0194 (17)	0.0016 (14)	0.0007 (14)	0.0039 (14)

### Geometric parameters (Å, º)

**Table:**

Br11—C5	1.902 (3)	C19—C24	1.401 (5)
O13—C12	1.198 (4)	C20—C21	1.382 (5)
O14—C12	1.346 (4)	C21—C22	1.384 (5)
O14—C15	1.466 (4)	C22—C23	1.391 (5)
N1—C2	1.365 (4)	C23—C24	1.383 (5)
N1—C10	1.324 (4)	C3—H3	0.9300
C2—C3	1.416 (5)	C4—H4	0.9300
C2—C7	1.423 (5)	C6—H6	0.9300
C3—C4	1.361 (6)	C9—H9	0.9300
C4—C5	1.400 (6)	C15—H15A	0.9700
C5—C6	1.368 (4)	C15—H15B	0.9700
C6—C7	1.417 (4)	C16—H16A	0.9600
C7—C8	1.435 (4)	C16—H16B	0.9600
C8—C9	1.370 (4)	C16—H16C	0.9600
C8—C12	1.495 (5)	C17—H17	0.9300
C9—C10	1.414 (5)	C18—H18	0.9300
C10—C17	1.476 (4)	C20—H20	0.9300
C15—C16	1.501 (6)	C21—H21	0.9300
C17—C18	1.324 (5)	C22—H22	0.9300
C18—C19	1.469 (4)	C23—H23	0.9300
C19—C20	1.404 (4)	C24—H24	0.9300
			
C12—O14—C15	115.8 (2)	C19—C24—C23	121.4 (3)
C2—N1—C10	118.0 (3)	C2—C3—H3	120.00
N1—C2—C3	116.8 (3)	C4—C3—H3	119.00
N1—C2—C7	124.0 (3)	C3—C4—H4	121.00
C3—C2—C7	119.3 (3)	C5—C4—H4	120.00
C2—C3—C4	121.0 (4)	C5—C6—H6	121.00
C3—C4—C5	119.0 (3)	C7—C6—H6	121.00
Br11—C5—C4	118.5 (2)	C8—C9—H9	119.00
Br11—C5—C6	118.6 (3)	C10—C9—H9	119.00
C4—C5—C6	122.8 (3)	O14—C15—H15A	109.00
C5—C6—C7	118.9 (3)	O14—C15—H15B	109.00
C2—C7—C6	119.0 (3)	C16—C15—H15A	109.00
C2—C7—C8	116.5 (3)	C16—C15—H15B	110.00
C6—C7—C8	124.5 (3)	H15A—C15—H15B	108.00
C7—C8—C9	118.1 (3)	C15—C16—H16A	109.00
C7—C8—C12	121.9 (3)	C15—C16—H16B	109.00
C9—C8—C12	120.0 (3)	C15—C16—H16C	109.00
C8—C9—C10	121.3 (3)	H16A—C16—H16B	110.00
N1—C10—C9	122.1 (3)	H16A—C16—H16C	110.00
N1—C10—C17	119.3 (3)	H16B—C16—H16C	109.00
C9—C10—C17	118.6 (3)	C10—C17—H17	118.00
O13—C12—O14	122.9 (3)	C18—C17—H17	118.00
O13—C12—C8	126.0 (3)	C17—C18—H18	117.00
O14—C12—C8	111.0 (3)	C19—C18—H18	117.00
O14—C15—C16	110.9 (3)	C19—C20—H20	120.00
C10—C17—C18	124.6 (3)	C21—C20—H20	120.00
C17—C18—C19	126.6 (3)	C20—C21—H21	120.00
C18—C19—C20	122.9 (3)	C22—C21—H21	120.00
C18—C19—C24	118.9 (3)	C21—C22—H22	120.00
C20—C19—C24	118.1 (3)	C23—C22—H22	120.00
C19—C20—C21	120.4 (3)	C22—C23—H23	120.00
C20—C21—C22	120.6 (3)	C24—C23—H23	120.00
C21—C22—C23	120.2 (3)	C19—C24—H24	119.00
C22—C23—C24	119.3 (3)	C23—C24—H24	119.00
			
C15—O14—C12—O13	2.1 (4)	C2—C7—C8—C12	178.8 (3)
C15—O14—C12—C8	−178.5 (3)	C7—C8—C9—C10	1.9 (4)
C12—O14—C15—C16	−86.7 (3)	C12—C8—C9—C10	−178.6 (3)
C2—N1—C10—C17	−178.4 (3)	C7—C8—C12—O13	−0.6 (5)
C2—N1—C10—C9	−0.1 (4)	C9—C8—C12—O14	0.4 (4)
C10—N1—C2—C3	−179.6 (3)	C7—C8—C12—O14	179.9 (3)
C10—N1—C2—C7	0.2 (4)	C9—C8—C12—O13	179.9 (3)
C7—C2—C3—C4	−0.8 (4)	C8—C9—C10—N1	−1.0 (4)
C3—C2—C7—C6	1.0 (4)	C8—C9—C10—C17	177.3 (3)
N1—C2—C7—C6	−178.8 (3)	N1—C10—C17—C18	7.5 (5)
N1—C2—C7—C8	0.7 (4)	C9—C10—C17—C18	−170.9 (3)
N1—C2—C3—C4	179.0 (3)	C10—C17—C18—C19	175.3 (3)
C3—C2—C7—C8	−179.5 (3)	C17—C18—C19—C20	16.4 (6)
C2—C3—C4—C5	−0.2 (4)	C17—C18—C19—C24	−161.1 (4)
C3—C4—C5—C6	1.1 (4)	C18—C19—C20—C21	−174.2 (4)
C3—C4—C5—Br11	−177.2 (2)	C24—C19—C20—C21	3.4 (5)
Br11—C5—C6—C7	177.4 (2)	C18—C19—C24—C23	174.4 (4)
C4—C5—C6—C7	−0.9 (4)	C20—C19—C24—C23	−3.3 (5)
C5—C6—C7—C2	−0.2 (4)	C19—C20—C21—C22	−1.3 (6)
C5—C6—C7—C8	−179.6 (3)	C20—C21—C22—C23	−1.1 (6)
C6—C7—C8—C9	177.8 (3)	C21—C22—C23—C24	1.2 (6)
C6—C7—C8—C12	−1.8 (4)	C22—C23—C24—C19	1.0 (6)
C2—C7—C8—C9	−1.7 (4)		

### Hydrogen-bond geometry (Å, º)

**Table:**

*D*—H···*A*	*D*—H	H···*A*	*D*···*A*	*D*—H···*A*
C6—H6···O13	0.93	2.22	2.848 (4)	124
C15—H15*A*···O13^i^	0.97	2.51	3.413 (4)	154

Symmetry code: (i) −*x*+1/2, *y*+1/2, *z*.
