# Supplementary figures and images for: Crystal structure of ethyl 6-bromo-2-[(E)-2-phenyl­ethen­yl]quinoline-4-carboxyl­ate
Source: Acta Crystallogr E Crystallogr Commun. 2015 Jan 17;71(Pt 2):o121. doi: 10.1107/S2056989014028266 (PMC4384604; doi:10.1107/S2056989014028266)

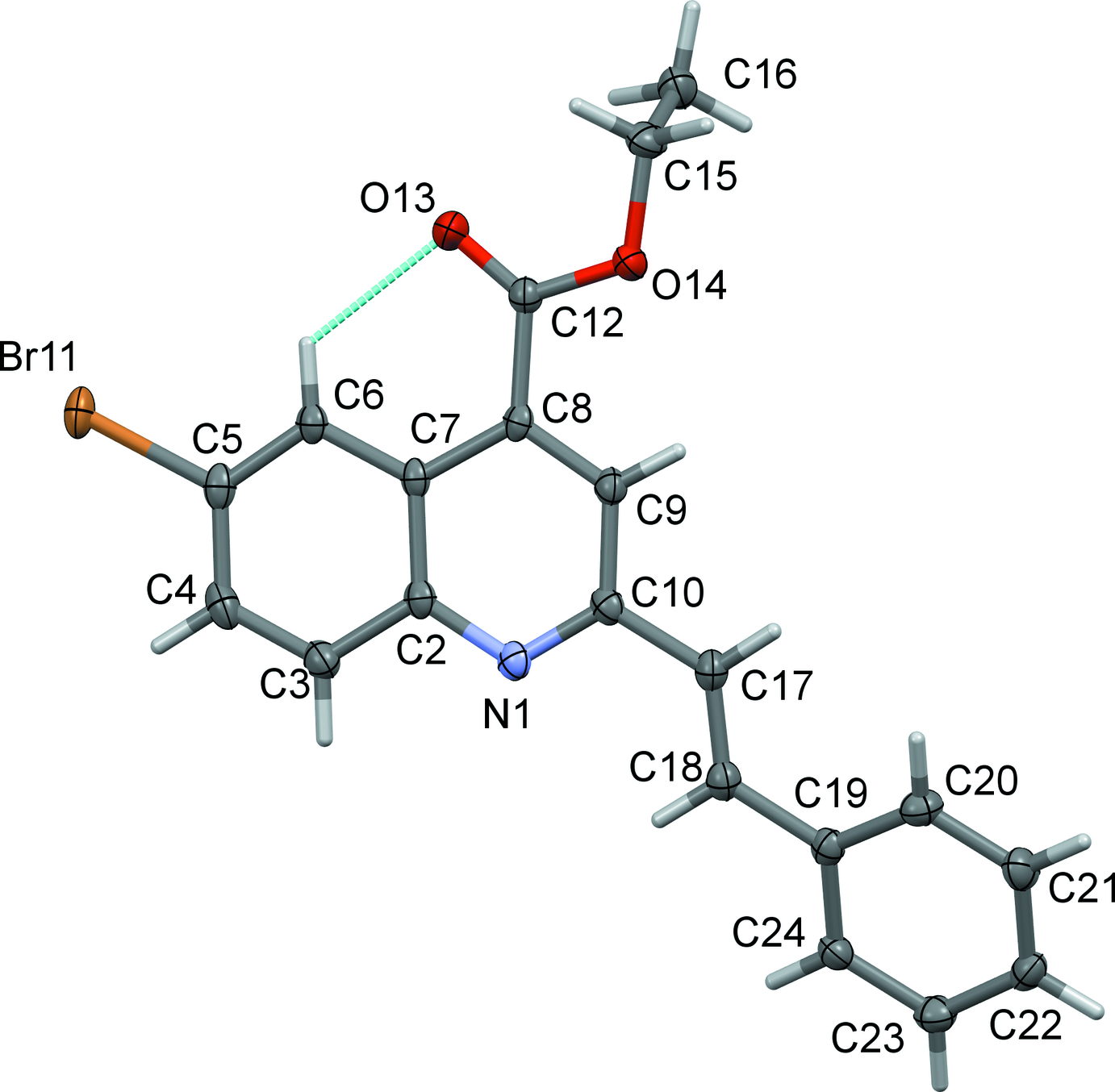

Supplement: Supplementary file 4 [file e-71-0o121-fig1.tif]

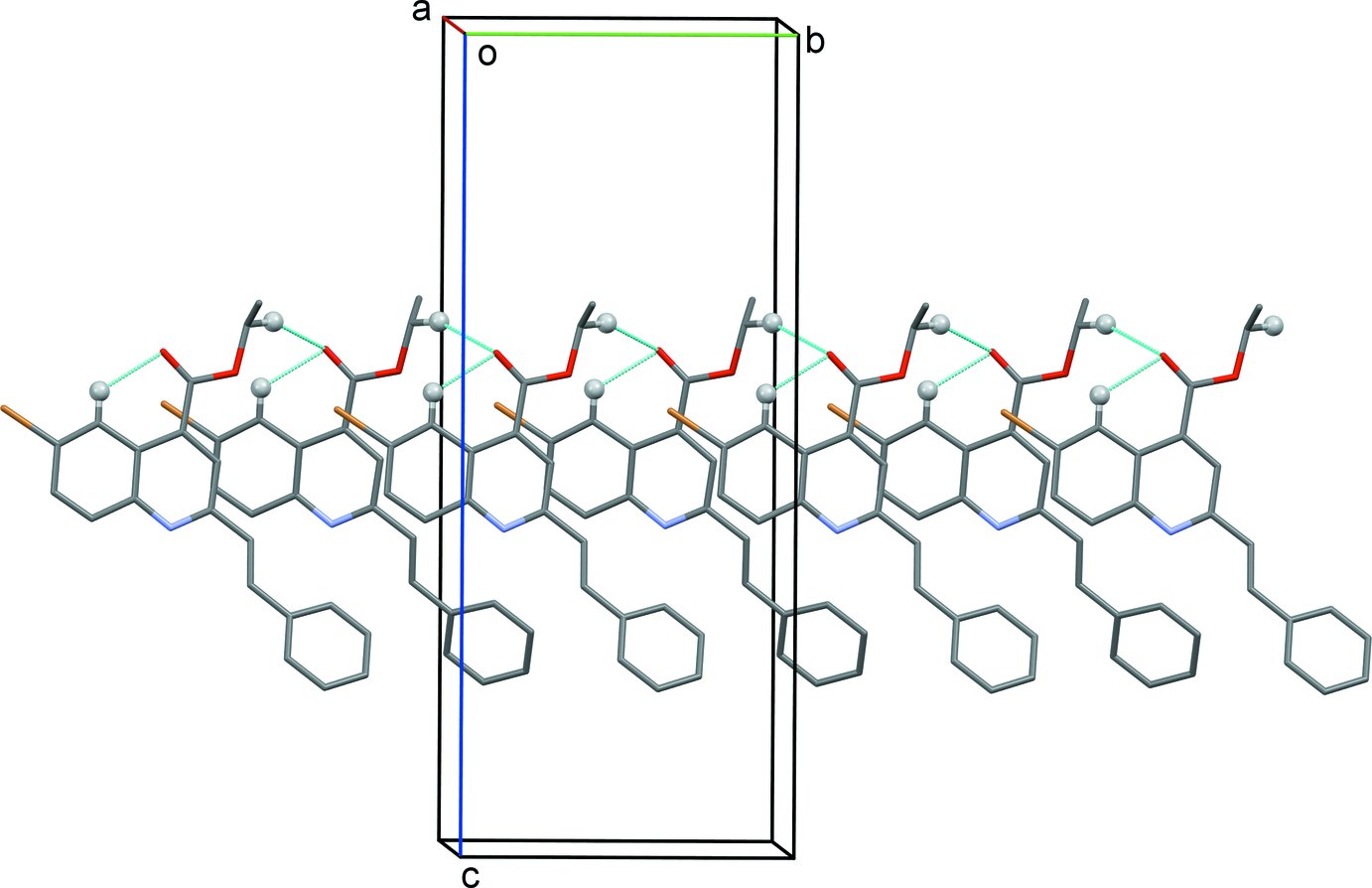

Supplement: Supplementary file 5 [file e-71-0o121-fig2.tif]
